# Minimal invasive open tibial fracture model in mice

**DOI:** 10.1007/s00264-025-06644-8

**Published:** 2025-08-30

**Authors:** Stefanie Hoelscher-Doht, Marietta Herrmann, Takahiro Higuchi, Klara Lill, Andrea Ewald, Rainer Meffert, Sebastian Haeusner, Mila M. Paul

**Affiliations:** 1 Department for Trauma, Hand, Plastic and Reconstructive Surgery, University Hospital, Würzburg, Germany; 2 IZKF Junior Research Group Tissue Regeneration in Musculoskeletal Diseases, Bernhard- Heine-Center for Locomotion Research, Würzburg, Germany; 3 Department of Nuclear Medicine, University Hospital, Würzburg, Germany; 4 Department for Functional Materials in Medicine and Dentistry, University Hospital, Würzburg, Germany

**Keywords:** Mouse, Mice, Fracture, Bone, Pin, Minimal, Invasive

## Abstract

**Purpose:**

Fracture models in animals are essential to analyze bone healing in musculoskeletal research fields. Especially in small animals, fractures are difficult to simulate and stabilize. Therefore, a fracture model is desirable with a short operation time, high safety of the model without stabilization failure and low costs. Aim of this study is the evaluation of a new open tibial shaft model in mice for musculoskeletal research.

**Methods:**

In 12 eight week-old wild type mice, an open tibial shaft fracture was simulated and stabilized with a retrograde over the fracture inserted intramedullary pin. X-rays confirmed the correct fracture localization and stabilization. After eight weeks of follow-up, the mice were euthanized. Fracture healing and biomechanical stability were analyzed in a micro-CT scan and in torsional load-to-failure tests.

**Results:**

The whole operations lasted in mean eight min and 50 s. All mice recovered very quickly after the operative intervention and started using the operated leg again on the first postoperative day onwards if not earlier. No infections or failure of the stabilization occurred. All fractures healed completely within 8 weeks and substantial callus formation was confirmed in the micro-CT analysis. Biomechanically, higher torsional moment and stiffness were found for the operated tibia compared to the non-operated tibia in the same mouse.

**Conclusion:**

The presented tibial fracture model with open osteotomy and retrograde pin insertion revealed minimal operative intervention and anesthesia, quick recovery and fracture healing with big callus formation. It is an easy to address fracture model for musculoskeletal research.

## Introduction

Mouse models are playing an increasingly important role in the analysis of basic scientific questions. Mice are used disproportionately often as laboratory animals in science and comprise over 70% of all animals used in translational and applied research [1, 2]. Murine fracture models are frequently used to study fracture healing. Due to the very small dimensions of the mouse, the long tubular bones are generally best suited for fracture models. Various models for fracture simulation on the femur and tibia have been described in the literature: *Brady et al. 2016* described a model of closed tibial fracture with insertion of a wire into the tibia below the knee joint and subsequent fracturing of the bone via a three point bending test with the wire in place [3]. A similar model of a closed tibial shaft fracture was described by *Schindeler et al. 2008*, to which other studies refer [4]. An alternative model is to osteotomize the femur and to stabilize the femoral shaft fracture by a plate osteosynthesis [5] or an external fixator [6, 7]. However, there is consensus in the field that existing preclinical models need optimization and that a limited number of well-characterized and clinically relevant models would be desirable [8].

Recent studies demonstrated that a tibial shaft fracture in a mouse model can be adequately stabilized using intramedullary osteosynthesis with a metal pin, leading to timely healing of the fracture: *Mori et al. 2021*, for example, showed that intramedullary fixation using TiNbSn enables good fracture healing [9]. *Casanova et al. 2021* analyzed the healing of a tibial fracture after stabilization with a “stainless-steel insect pin”, which was inserted antegrade from the knee joint into the bone like described by *Schindeler et al. 2008*, by micro-CT [10]. Three weeks postoperatively, *Casanova et al. 2021* were able to demonstrate the largest callus formation on CT. Remodeling of the callus occurred histologically and morphologically in the CT scan six weeks postoperatively [10].

All the studies mentioned, exhibited some negative aspects, such as long operating times with a highly invasive procedure [11]. These two factors are closely linked in that extensive preparation for fracture stabilization using internal plate osteosynthesis at the femur takes time and also traumatizes the surrounding tissue considerably more [11]. Both ultimately increase not only the overall traumatic aspect for the test animals, but also the risk of perioperative complications such as wound healing disorders, post-operative bleeding, infections, etc. In the tibia, the extreme bending of the part of the bone close to the knee joint can very easily lead to malpositioning of the stabilizing wire, which is traditionally inserted intramedullary antegrade from the knee joint [3]. An intraoperative X-ray position verification, as is usually carried out during fracture treatment in humans, is normally not possible in animal experiments. A malposition would be visible at the earliest after the operation has been completed and X-rays have been taken, although X-rays in mice alone cannot reliably rule out a malposition. The generation of two planes or dynamic fluoroscopy to verify the correct position of the wire in the bone is not possible in mice due to the X-ray technology in laboratory animal facilities.

The aim of this study was to establish a fracture model of the tibia that is fast and easy to perform in order to minimize the invasiveness of the procedure, shorten the operation time and quick regeneration of the animals after anesthesia. Therefore, in this study we present an alternative surgical technique to the previously described antegrade intramedullary stabilization of the tibia: A decisive advantage of the tibial fracture model in our study is that the stabilizing wire (stainless-steel insect pin, see above) is only inserted after the open tibial shaft osteotomy has been performed, thus avoiding any complications with the wire (such as unintentional breaking or bending) or malpositioning due to insertion via the fracture gap. Another advantage is that this model minimizes the invasiveness of the operation, as only a small surgical field is required and little surrounding tissue needs to be prepared, which simultaneously reduces the rate of postoperative complications. Particularly in the knee joint, traumatizing steps such as the accidental opening of the joint or potential injury to the patellar tendon when reaming the tibia and inserting the wire are eliminated. Fracture healing was verified using micro-CT analysis and the callus was quantified. The stability of the fractured bone was also analyzed biomechanically.

## Materials and methods

### Animals

For the presented study, 12 mice of the line C57BL/6J (JAX™ Mice Strain) were bought from Charles River (Research Models and Services, Sulzfeld, Germany) at the age of six weeks. The six female and six male mice were kept for two weeks in the animal facility of the Department of Functional Materials in Medicine and Dentistry, University Hospital Würzburg. Animals were allowed an unrestricted supply of food and fluids. The study received a positive ethics vote from the local committee of the government of Lower Franconia (RUF-55.2.2-2532-2-1518-41).

### Surgical method

**Fig. 1:**
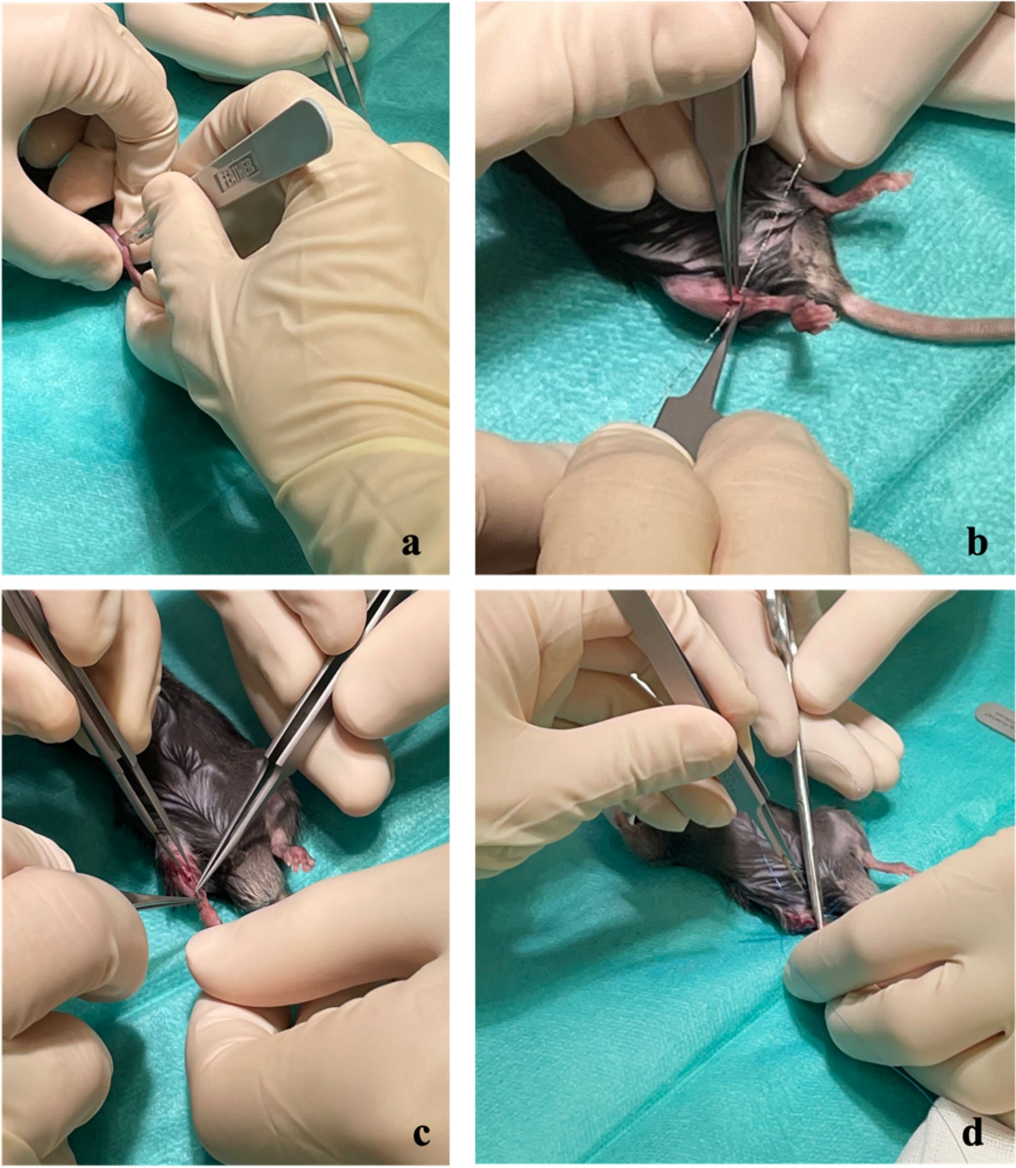
The various surgical steps are shown: After skin incision (**a**), the tibia has been carefully circled and osteotomized with a Gigli saw at the transition area from the proximal to the middle third (**b**). A 0.2 mm pin was inserted retrograde over the fracture and then the distal tibial fracture fragment was reduced by the wire (**c**). In the end, the skin was sutured intracutaneously (**d**)

The eight week-old mice with an average weight of 18.88 g for females and 26.26 g for males were prepared for the operations: In the scruff of the neck of the mice, Tramadol (Tralieve, 25 mg/kg) was administered subcutaneously (s.c.) for analgesia. After placing the animals on a heating plate, an ophthalmic cream was applied and an inhalation anaesthesia with continuous supply of Isofluran was started. After shaving the surgical area of the right leg, it was washed with disinfectant, covered with a sterile drape, and a short longitudinal incision was made in the skin on the lateral side of the right middle third of the lower leg. After separating the tibialis anterior muscle from the bone with a raspatory, the tibia was carefully bypassed medially and laterally. While protecting the soft tissue, a transverse shaft fracture was then created in the transition area between the proximal and middle third using a Gigli saw. The proximal part of the fracture was then threaded on retrograde with a 0.2 mm pin. This was pushed into the proximal tibia without damaging the knee joint or the patellar tendon. Distally, the fracture was threaded over the wire and the wire was pushed back into the proximal part of the fracture until it was anchored in the area of the ankle joint. The wire was thus securely positioned intramedullary and did not protrude into the proximal part. After irrigation, an intracutaneous skin suture was applied. A spray dressing for skin closure was used in the end. Hydration was ensured by intraperitoneal injection of saline (1000 µl). The operations were performed under loupes using a microsurgical technique by two experienced orthopedic surgeons (M.M. Paul and S. Hoelscher-Doht). Postoperatively, the fracture and pin-stabilization were confirmed by conventional radiograms (Faxitron™ Trident^®^, Hologic, Marlborough, MA, USA). All surgical steps and postoperative x-rays are demonstrated in Fig. 1.

For postoperative analgesia, the mice were offered a liquid solution containing tramadol (Tramadol 0.2 mg/ml + in sweetened autoclaved water) for five days. After the operation all mice were kept in groups of two together in the animal facility. Male and female mice were kept separately. A scoresheet was used to closely monitor wound healing, behaviour, and weight progress (Table 1). After three and six weeks, conventional radiograms were obtained in short anesthesia by Tramadol s.c. and Isofluran as described above during operation. Eight weeks after operation, the mice were euthanized, and both legs were removed and carefully dissected for further analysis.

### Scoresheet

**Table 1:** The follow-up of the operated mice was documented based on the scoresheet, to detect potential stress and to sum up the stress points carefully. Grades and probable consequences are listed

	Potential Points of stress	
Body weight	0–20	
General health situation	0–20	
Behavior	0–20	
Wound healing	0–20	
Abnormal tibial fracture	0–20	
**Sum of stress points**	**Grade of distress**	**Consequence**
0	none	none
1–9	low	daily control check-up
10–19	middle	veterinarian check-up twice daily
> 20	high	termination of the test

### Groups for imaging and biomechanics

To analyze fracture healing, callus formation and biomechanical torsional stability of the bone, the non-operated tibiae (*n* = 12) (Group 1) were compared to the operated tibiae in the same mice (Group 2) (12 mice, per group 6 female / 6 male tibiae, Table 2). As a control group, tibiae from wild type mice were used (Group 3) (10 mice, 4 female / 6 male). The control group comprised animals from other scientific research groups that were made available to us for organ removal after euthanaesia. The organ removal (tibial bone) was reported to the government of Lower Franconia in accordance with the animal welfare guidelines.

**Table 2:** Non-operated tibiae (group 1) were analyzed using micro-CT scans and biomechanically with the matched tibia from the operated side of the same mouse (group 2). Both groups were also compared with a control group of tibiae in wild type mice (group 3)

Group	Female	Male	Leg	Operated Mouse	Age at Euthanasia	Genetic Type
**1**	6	6	Non-Operated	+	16 weeks	wild type
**2**	6	6	Operated	+	16 weeks	wild type
**3**	4	6	Non-operated	-	16 weeks	wild type

### Micro-CT analysis

**Fig. 2:**
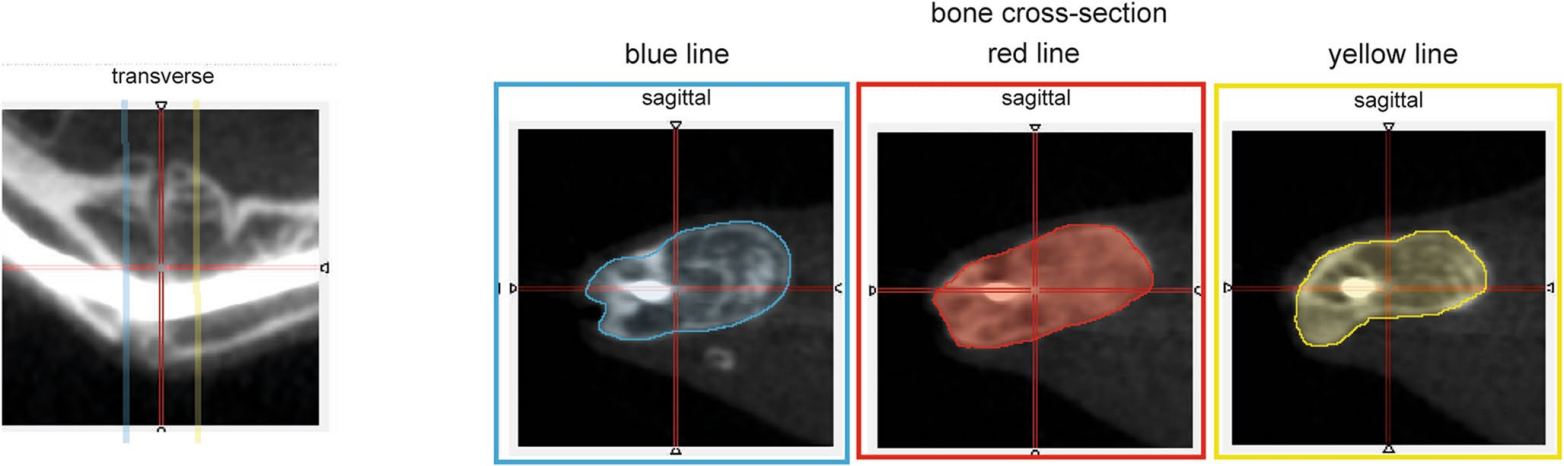
Callus formation was calculated by measuring the bone cross-sections in 0.3 mm slices anterior and posterior (blue and yellow lines) at the fracture (red line) in the transverse views (left side) of the micro-CT scans

Regions of interest (ROI) were specified in the transverse view of the micro-CTs at the healed fracture line and on bone cross-sections in 0.3 mm slices anterior and posterior. The sum of the areas of each ROI was defined as the callus volume (total area of blue + red + yellow) (Fig. 2) [12]. Additionally, fracture healing was assessed in all three views (transverse, sagittal, axial) with a region of interest of +/-0.3 mm in each direction [13, 14]. The micro-CT scans were acquired using the MiLabs U-SPECT5-E system (MiLabs, Utrecht, Netherlands), applying the standard high-resolution protocol and reconstructed with an isotropic voxel size of 30 μm (0.03 × 0.03 × 0.03 mm³). The data was evaluated by the Amide Software Version 10.9 (SourceForge Headquarters, San Diego, USA). 3D-imaging was performed with OsiriX lite software 14.1.1 (Pixmeo SARL, Bernex, Switzerland).

### Biomechanical tests

The tibiae were dissected from soft tissue and embedded in a custom-made device for the tests. The pin in the operated bones was left in place, because removing would have been of high risk of damaging the tibiae too much for veritable biomechanical testing. Both ends of the bones, proximal and distal of the tibial bone were fixed with Palacos^®^, Heraeus Medical GmbH, Wehrheim, Germany, bone cement for implantation of endoprosthesis. After 30 min of hardening, the embedded tibia with a length of 7.5 mm was placed in a custom-made device in a material testing machine Zwick Roell 2.5kN. In a load-to-failure test, the bones were tested with torsional loading with a crosshead speed of 10 mm/min until a spiral fracture occurred (Fig. 1). The maximum load was recorded by the software testexpert III-V1.61. Maximum torque [Nmm] and torque stiffness [N/mm] were calculated by considering the diameter of the bone measured in the micro-CT. The biomechanical testing underwent bones of three groups: Group one comprised the non-operated side of the experimental mice (*n* = 12, 6 female, 6 male). In group two, the operated tibiae of the experimental mice were tested (*n* = 12; 6 female, 6 male). In group three, 14-week-old wild-type mice were tested as a control group. Both tibiae of the six male and four female mice were dissected and underwent biomechanical testing (*n* = 20).

### Statistics

All groups underwent an analysis for normal distribution by a Shapiro-Wilk test. Significant differences were further calculated by a t-test or when the data were not normally distributed by a Kruskal-Wallis Test. The statistical analysis was done in consultation with the statistical service of the Institute of Clinical Epidemiology and Biometry at the University of Würzburg, Germany. In box plots, horizontal lines represent median, boxes quartiles and whiskers tenth and 90th percentiles. Crosses indicate mean values. Asterisks indicate significance level (* *p* < 0.05).

## Results

### Animal care

All mice survived the surgery and recovered very quickly. After just 30 min, they were climbing the cages all over again. The operation time lasted on average eight min and 50 s ± 2 min 21 s. During the first eight weeks after surgery, the stress and behavior of the mice were followed up and none of the mice showed signs of distress or suffering from discomfort. None developed local wound healing problems, and all passed the eight weeks of observation period time. All mice developed a minimal reduction of weight during the first days after operation and therefore scored 1point in the scoresheet. Within a very short time, they gained weight again (within 3–7 days, mean 5.66 days).

### X-ray analysis and micro-CT

**Fig. 3:**
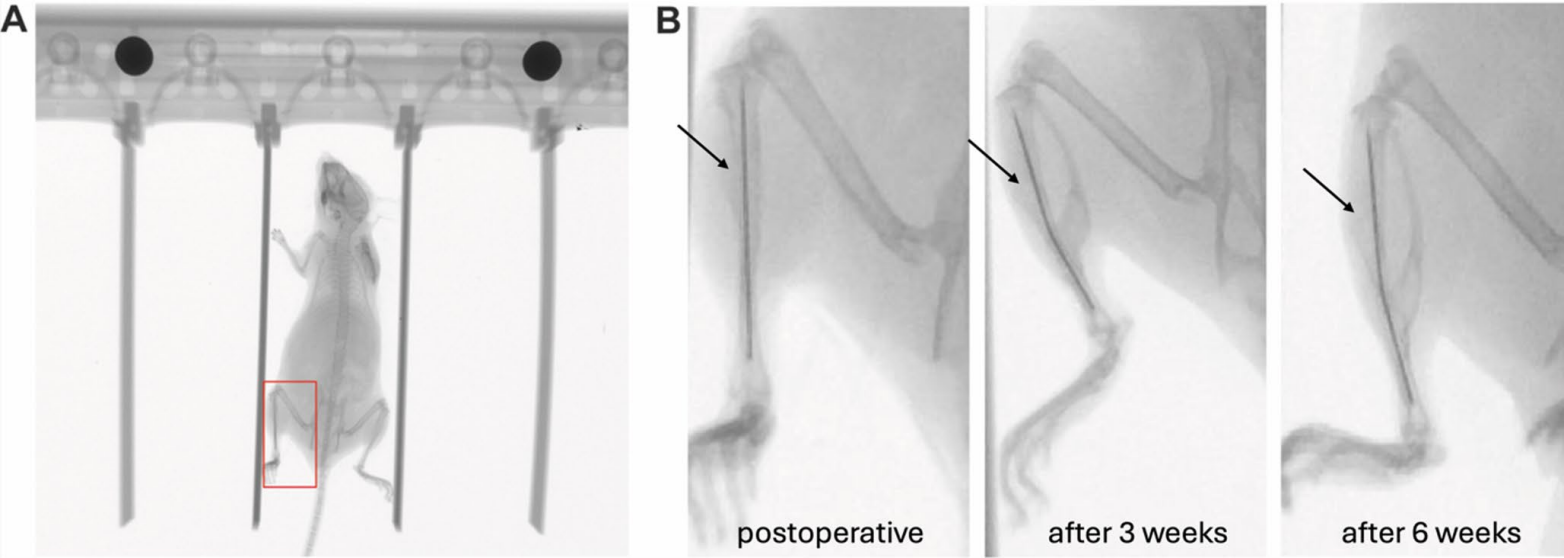
(**A**) X-rays were conducted in anesthetized animals to visualize fractured tibiae (marked with red box), position of the inserted intramedullary pin as well as fracture healing. (**B**) Example x-ray of a fractured tibia (highlighted from red box in A) immediately after the operation and 3 and 6 weeks postoperatively

Postoperative x-rays confirmed a correct intramedullary implant position and fracture generation in the zone of transition from the proximal to the middle third of the tibia in all 12 operated mice. After three weeks, initial fracture healing with callus formation could be demonstrated in all cases. Although the fracture gap was conventionally visible radiologically after 6 weeks, a distinctive callus formation could already be detected after this time, which suggests complete healing after this period. Figure 3 shows an example of the postoperative x-ray images and the x-ray controls after three and six weeks in a female mouse.

**Fig. 4:**
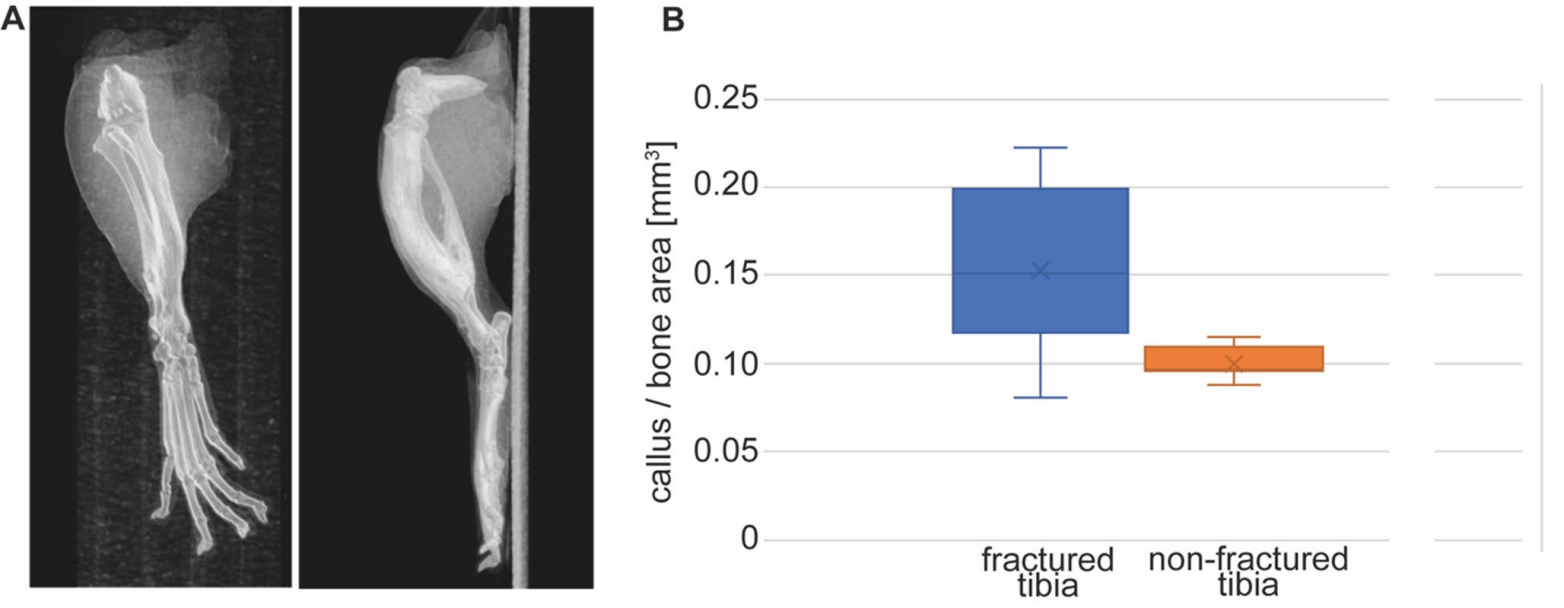
The micro-CT scan of the legs revealed a complete bone healing with callus formation. (**A**) 3D visualization giving an impression of the bone fracture healing of the fractured tibiae. (**B**) Comparison of the bone volume of the fractured tibia (blue) and at the same bone level of the non-fractured leg of the same mice (orange) demonstrates a high bone formation

Micro-CT analyses revealed a complete healing of the fracture after eight weeks with big callus formation (Fig. 4A). In total, 0.3 mm^3^ around the fracture, the operated legs demonstrated a bone tissue of 0.15 mm^3^ ± 0.05 mm^3^. The micro-CT scan of the non-operated leg of the same mice was analyzed at the same region of the tibiae like the operated one and a mean value of 0.10 mm^3^ ± 0.01 mm^3^ was calculated (Fig. 4B).

### Biomechanics

**Fig. 5:**
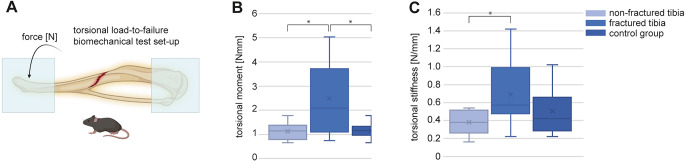
(**A**) In a material testing machine Zwick Roell 2.5kN, the tibial bone underwent a torsional Load-to-Failure test. Both ends, proximal and distal were embedded in a custom-made device and the middle third of the bone was loaded till a spiral fracture occurred. Torque to failure [Nmm] and torque to stiffness [N/mm] were determined (created with biorender). (**B**, **C**) Mean values for the torsional moment (**B**) and torsional stiffness (**C**) in non-fractured tibiae, fractured tibiae and control legs. * Asterisk marks *p* < 0.05

In the biomechanical torsional tests, the operated tibiae revealed a higher torsional moment (2.50 ± 1.52 Nmm) than the opposite legs of the same mice (1.14 ± 0.35 Nmm; *p* = 0.02), and of the tibiae of the control group with wild type mice in the same age (1.17 ± 0.25; *p* = 0.02) (Fig. 5B). For the torsional stiffness higher scores were determined for the group of operated legs (0.69 ± 0.36 N/mm) compared to the opposite leg of the same mice (0.38 ± 0.13 N/mm; *p* = 0.01). No differences were found for the operated legs compared to the control group (0.51 ± 0.24 N/mm; *p* = 0.22) (Fig. 5C).

## Discussion

Fracture models in animals are essential for studying various fracture healing aspects in musculoskeletal research [11]. Mice are the most common species in several countries used in animal studies for human medical science. Mouse fracture models include the femur and tibia. However, the currently described models have limitations in terms of invasiveness and subsequent complications. Therefore, the aim of this study was to establish a minimally invasive fracture model on the tibia, in which the fracture can be safely set without major soft tissue trauma, and a subsequent secure stabilization of the fracture can be achieved. Fortunately, all animals survived the operation and the following healing process without complications. The mice recovered very quickly after the short operation time of eight min on average (5–12 min range) and were already using the operated leg fully again the following day when weighed. The surgical technique ensures the intramedullary position of the wire without any risk of extramedullary malpositioning. As in humans, we consider intramedullary stabilization to be a very good anchoring of a shaft fracture of the long tubular bones, which heals well under load. In contrast, plate osteosyntheses on the femur can lead to plate failure or plate pull-out. A further advantage of the model presented here is that the retrograde insertion of the wire into the tibia and the pronounced bending of the proximal third on the mouse mean that no accidental injury to the knee joint or the patellar tendon can occur. In our own preliminary tests for this study, we were able to verify the difficulties in antegrade threading of the tibia: Particularly when opening the medullary canal proximally, injury to the patellar tendon by the reamer happens quickly.

One limitation of the model for the biomechanical examinations is certainly the inability to easily remove the wire due to the lack of protrusion out of the bone as described by other authors for antegrade threading. In principle, the sole intramedullary wire in the tibia is negligible in torsion testing. However, significantly higher torsional moments were demonstrated in the operated tibiae with pin compared to the non-operated tibiae of the same mice and compared to the control mice. This higher stability can be explained on the one hand by the large callus formation and on the other hand by the potentially clamped intramedullary pin. Nevertheless, it is crucial for the model that all fractures healed, as demonstrated by micro-Ct. In all animals, the postoperative follow-up was uneventful with timely weight gain. None of the animals showed any unusual behavior. In particular, the rapid recovery of the mice after surgery was impressive. We associate this with the very short anesthesia and operation time.

In the past, it has been repeatedly discussed in the literature whether locking of the nail is necessary for intramedullary stabilization of a femur or tibia fracture in mice. The authors tend to see the disadvantages of such a technique as longer operating time, technically very demanding procedure, questionable correct position of the locking screw in the nail and bone with very limited possibilities of intraoperative control in contrast to humans. The advantage, the rotational stability of the fracture, is negligible in the mouse from our point of view, as a rotational failure in the mouse is not clinically comparable to the clinical impairments in humans. Full weight bearing of the mouse leg was possible without restriction in our study.

## Conclusions

The model presented in this study, a minimally invasive facture of the tibia in mice with retrograde threading over the fracture and stabilization with a pin, represents an interesting alternative to existing models: on the one hand, the test animals recover quickly after the very short operation time. On the other hand, the model is cost-effective due to the use of a pin instead of plates or blocking intramedullary nails specially produced for mice. Moreover, all fractures healed after eight weeks without the death of an animal.

## Data Availability

All data is provided in the main text.
